# Cases of Varioloid Eruption Occurring Subsequent to Vaccination

**Published:** 1824-04

**Authors:** R. Wade

**Affiliations:** Member of the College of Surgeons, and Apothecary to the Westminster General Dispensary.


ART. VIII.—*Cases of Varioloid Eruption occurring subsequent to Vaccination.* By R. WADE, Member of the College of Surgeons, and Apothecary to the Westminster General Dispensary.

THE question as to the comparative security which vaccination affords against small-pox, has lately been much agitated, and the public much alarmed by constant reports of cases of small-pox having occurred notwithstanding previous vaccination. The consequence of these reports has been, that the prejudices against vaccination have been strengthened; whilst those in favour of inoculation have, at the same time, gained ground, more especially amongst the uninformed; and those practitioners who still inoculate have received encouragement in spreading small-pox, and thus counteracting the good effects of vaccination. Almost every eruption arising from the variolous infection has been called small-pox. That cases of true small-pox may occasionally happen after vaccination, there can be but little doubt; yet ought these cases to be considered as depreciating the value of vaccination? Certainly not. There are but few, if any, remedies which can be deemed infallible; besides, instances sometimes occur where the constitution is completely insusceptible of the vaccine disease, although, from its producing a sore arm, parents may flatter themselves that their children are secure from small-pox. If inoculation be justifiable in any instance, it is in that where the constitution is insusceptible of vaccination, and where it has no other effect than perhaps to produce a little redness of the arm. The merits of vaccination rest on too firm a basis to shrink from investigation. If all the cases of eruptions which are called small-pox after vaccination were brought forward, and impartially stated, (which is the only way to arrive at truth,) I believe that most of them, like those which are to be related in this paper, as well as in every case that I have seen, would be found so modified by previous vaccination, that they would rather tend to increase than to diminish our estimation of this most valuable discovery.

On Wednesday evening, February 25th, I was requested to see a friend of mine, who feared that he had small-pox. I found him with a slight degree of fever, attended by an eruption of papulæ, chiefly in the face and hands; some of them containing a fluid of a light yellow colour, with no depression in their centre. As the gentleman was a lawyer, perhaps I cannot do better than give his own brief: his description of symptoms previous to my seeing him, was as follows:—“A little boy, who sleeps in the next room to mine, is just recovering from small-pox. On Saturday evening last, ten days after the eruption appeared on him, I was attacked with violent pain in the front and back part of my head, with considerable drowsiness, sickness, and a total loss of appetite. On the Tuesday morning, some pimples appeared on my face and hands; afterwards, a few on other parts of my body.” After the appearance of the eruption, the feverish symptoms subsided, and they were very slight when I saw him. He had taken some aperient medicine, which was ordered to be continued. On Friday evening, when I again saw him, the fluid in the eruptions which first appeared had been nearly absorbed; they had now the resemblance of dark red spots, very little raised, and were reduced to about one-third of their previous size. A few others had then appeared, which have since taken a similar course; appearing gradually to subside after the third day.

This gentleman had been vaccinated, and has two marks left by it on his arm.

I, of course, felt anxious to ascertain the nature of the eruption which had appeared in the boy, and the following is the history given me by his mother:—He is fifteen years of age, and was vaccinated, when between three and four months old, immediately from the arm of another child; had one vesicle, the scar of which is still remaining. This boy had been visiting a relation, who had small-pox, of a very mild kind, from inoculation. About a week after his first being exposed to the contagion, he complained of sickness and pain in the head, with little fever, but not sufficient to keep him from school. Four days afterwards, an eruption of small pimples appeared; this was on a Monday. The Thursday following, the eruption had a yellowish appearance; on the Saturday, a brown speck was observed in the centre; and, from this time, the eruption has been gradually dying away: it has now the appearance of small branny spots. Very little fever accompanied the disease.

Another of the family, a little girl, eleven years of age, was vaccinated when six months old, and had two vesicles; the scars of which are still very plain. About ten days after the eruption appeared on her brother, she complained of slight pain in the head, and was a little feverish; five papulæ came out on her face, which were followed by four on her right arm, three on the left wrist, and seven or eight on each leg. On the second day they were of a light yellow colour, having no depression in their centre; the fourth day, they assumed a reddish-brown hue, and from this time gradually diminished, having, on the seventh day, the appearance of very small dark-red spots.

This little girl's sister, aged seven years, was vaccinated when four months old; she had two vesicles, the scars are remaining. Only three papulæ appeared on her face, which pursued, as nearly as possible, the same course as her sisters, without any constitutional affection.

These cases were considered, by the mother of the children, as small-pox, and as such, of course, related to her friends; but how very different a course did they take to true small-pox, uninfluenced by previous vaccination: and I may here observe, that no ulceration occurred in any of the cases, although the boy had the eruption tolerably thick upon his face. It is worth inquiry whether one vaccine vesicle may be considered sufficient to secure the constitution from small-pox? I know that Mr. Carpue, surgeon to the vaccine establishment in Percy-street, is never satisfied with less than three; and certainly, in the cases just related, the boy, who had but one vesicle, was the only one who had an eruption of any consequence; though even in him the constitutional symptoms were slight.

*March*, 1824.

